# A systematic survey of RUM process parameter optimization and their influence on part characteristics of nickel 718

**DOI:** 10.1038/s41598-023-28674-1

**Published:** 2023-01-31

**Authors:** Dipesh Popli, Usha Batra, Velaphi Msomi, Shubham Verma

**Affiliations:** 1 GD, Goenka University, Gurugram, Haryana India; 2 Manav Rachna International Institute of Research and Studies (A Deemed to be University), Faridabad, Haryana India; 3 Mechanical Engineering Department, Cape Peninsula University of Technology, Cape Town 7535, Western Cape, South Africa; 4 MMEC, Maharishi Markendeshwar (Deemed to Be) University, Mullana, Haryana India

**Keywords:** Aerospace engineering, Mechanical engineering

## Abstract

This research is focused on the drilling of Nickel based super alloy with diamond metal core drill and identified the significant parameters of rotary ultrasonic machining that optimise the machining rate (MR) and surface quality. Four general parameters: workpiece material, workpiece thickness, tool material, and tool size; and four RUM parameters: tool rotational, feed rate, ultrasonic power rating, and abrasive grit size of the tool were tested against and surface quality of the cut. The results indicated that the maximum value of *MR* of 0.8931mm^3^/sec is acquired at higher level of tool rotation, feed rate, ultrasonic power and moderate level of abrasive grit size of diamond. The minimum surface roughness (*R*_*a*_) 0.554 µm is observed at higher level of rotational rotation, Moderate value of feed rate, ultrasonic power and diamond abrasive grit size. In addition, for single-objective and multi-objective functions, the particle swarm optimization (PSO) approach is used to find the optimum values for process parameters. Furthermore, a scanning electron microscope is also utilized to check the machined surface after RUM. It is concluded that microcracks are observed on the machined surface.

## Introduction

With the development of aircraft engine technology, composite and hard-to-cut materials are being used in the new engines more and more. This finding shows that there is a greater need for processing techniques and component capabilities for the machining of challenging materials.

A nickel based super alloys are unique class of metallic materials with a remarkable combination of elevated temperature strength, toughness, and resistance to deterioration in corrosive or oxidising conditions^1^.

**Figure 1:**
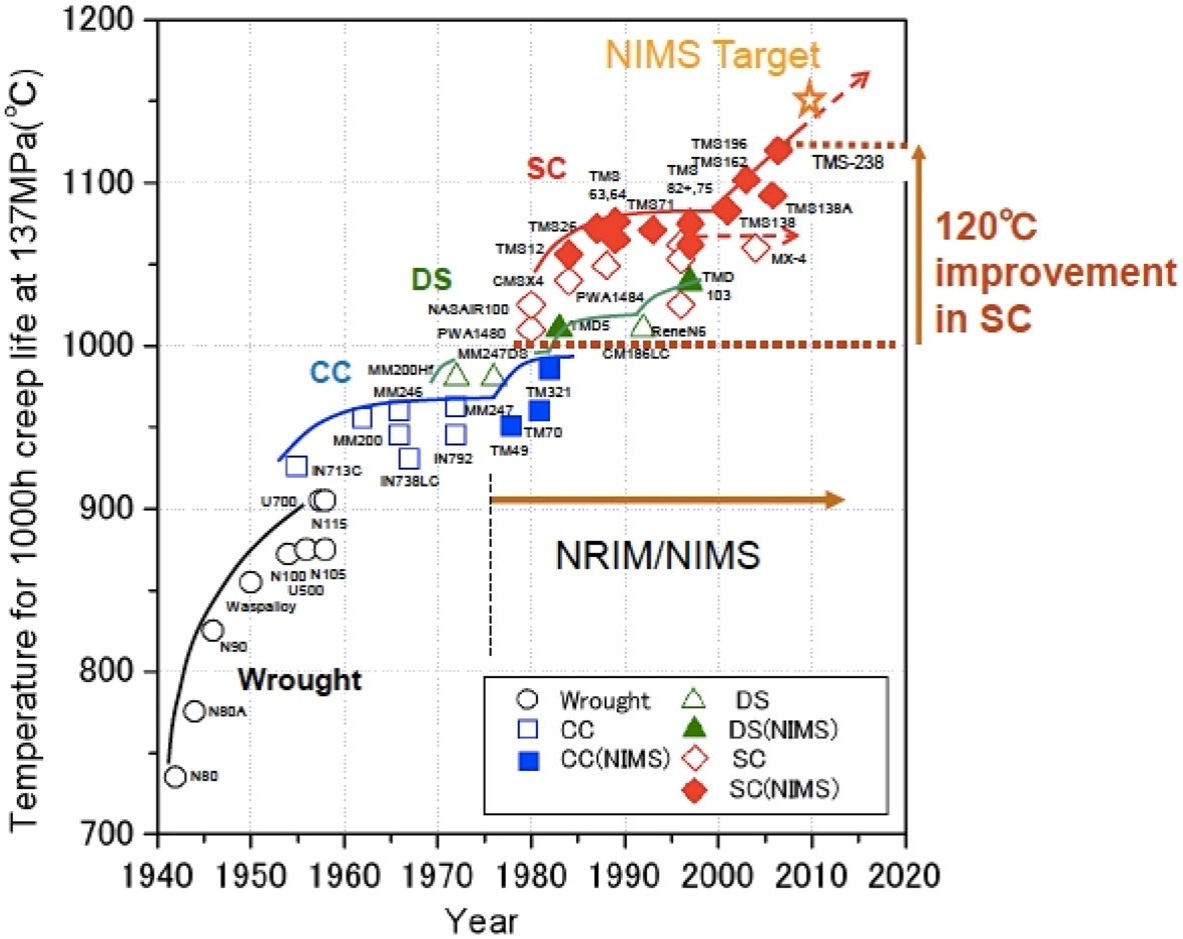
The development in creep rupture temperature capability of Ni-base superalloys under 1100 ℃—137 MPa^3^.

**Figure 2:**
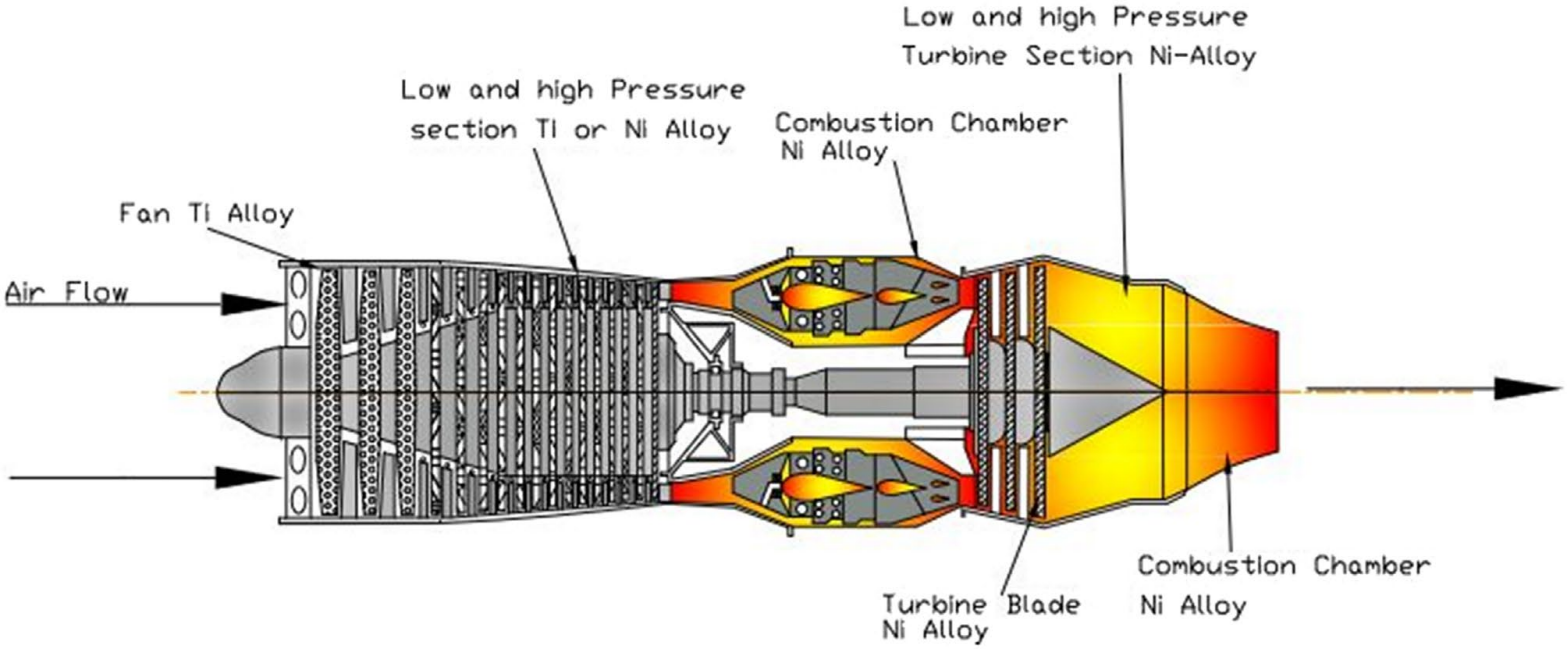
Utilises for nickel-based superalloys, which typically account for around 50% of a jet engine's weight.

Figure 1 shows the advancement in nickel based super alloy’s temperature capability which has increased year by year owing to the advanced processing, alloy development, use of the thermal barrier coatings, innovative and effective cooling schemes^2^. The components of aircraft engine, such as the casing, compressor discs, bearing ring, blades, turbine disc, and other parts operating in the high temperature, are made with nickel-based superalloys because of their high strength, strong corrosion resistance, excellent thermal fatigue properties, and thermal stability^3^. The numerous superalloys based on nickel that are used in jet engines are listed in Fig. 2.

Fifty percent parts of the jet engine is made by Inconel 718. Inconel is a Ni–Fe–Cr alloy^4^. However, the tensile strength Inconel 718 can reach 1393 MPa at room temperature. The component’s machining become hard due to its machinability. It has machinability only 8–20% of steel which leads to inefficient processing.

Additionally, the machining of nickel-based superalloys results in increased tool oxidation wear, adhesive wear, mechanical & diffusion wear, and, which reduces tool life. For instance, the rough and fine drilling of a nickel-based superalloy blade with a medium drilling length requires more time. For machining of super alloys, a frequent tool wear is considered to be the direct factor that limits the processing efficiency, while the sharp temperature rise caused by the heavily work-hardened surface being machined is a key factor to accelerate the tool wear^5^.

**Figure 3:**
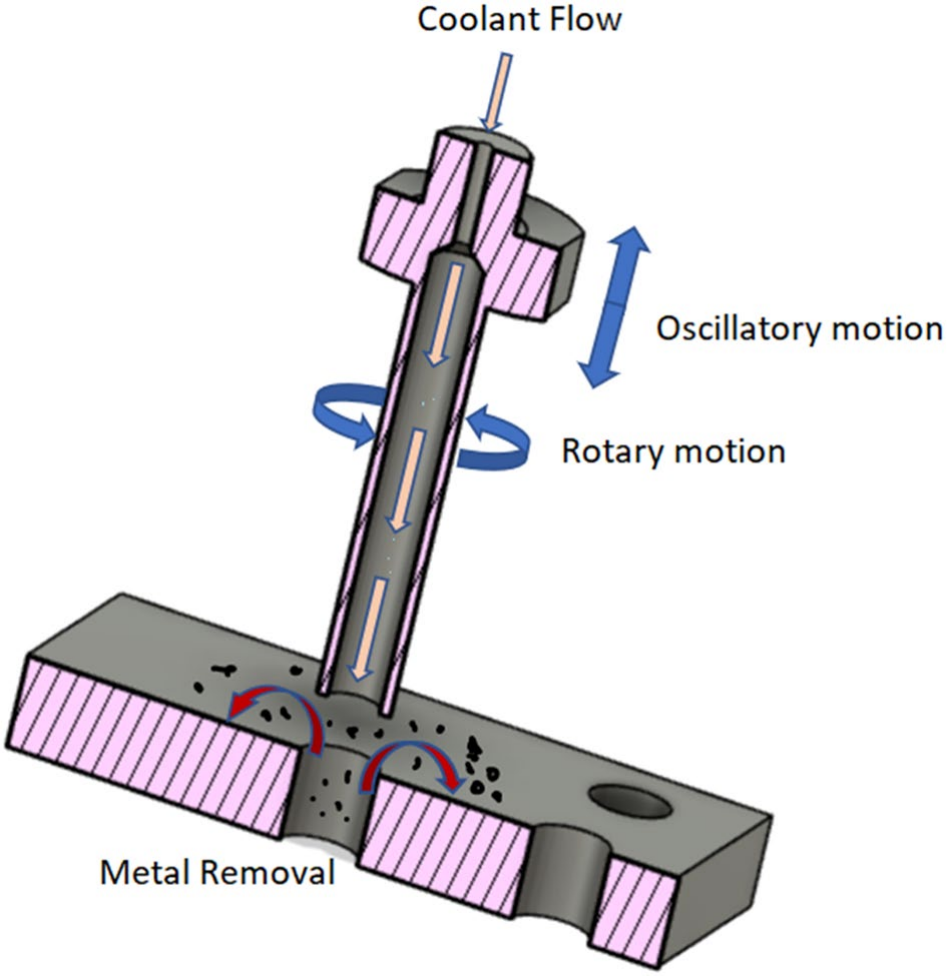
Process principal of RUM.

According to Habeeb et al.^6^, thermally induced cracking was the main reason for tool failure at high cutting rates. This happens as a result of the edges being subjected to a significant amount of thermal shock as a result of the high temperature brought on by fast cutting speeds and significant temperature change^7^. Conventional drilling is commonly faced with some difficulties due to heat localization in the cutting zone resulted by drill embedding in workpiece. The cutting temperature directly affects dimensional accuracy of drilled hole, surface quality, and tool life. Lofti et al. used ultrasonic assisted drilling under presence of nano-fluid minimum quantity lubrication for 1045 steel and found that due to reduction of friction coefficient caused by application of ultrasonic vibration, the wear mode of drilled surface is changed from adhesive type to abrasive one and formation of built-up edge is restricted that results in better surface finish^8,9^. Lofti et al. developed a mechanistic model of workpiece deflection for aluminium 7075. With ultrasonic assisted and without ultrasonic assisted drilling was performed on the workpiece. It was found that in both the experimental and theoretical approaches, with the increase in feed rate causes an increase in the deflection of the workpiece. This is due to the increase in thrust force values that was significantly influenced by feed motion^10^. Although super hard cutting tools like CBN and PCBN play some roles in improving the processing efficiency of nickel-based superalloys, ceramic cutting tools like alumina matrix and Si3N4 still play an important part. It is found that CBN tool is capable of machining of Inconel 718 as compared to carbide tool. In the present-day scenario, rotary ultrasonic machining (RUM) can be employed for machining of complex and tough structure material such as ceramic, titanium, glass, etc.^11^ Figure 3 indicates the processing method of RUM. A rotary core drill with metal-bonded diamond abrasives is ultrasonically vibrated and fed toward the workpiece at a constant feed rate or a constant force (pressure). Coolant pumped through the core of the drill washes away the swarf, prevents jamming of the drill, and keeps it cool. There are two mechanisms for the RUM process: firstly, by the process of ultrasonic vibration, material removal is done; secondly through the traditional diamond abrasive grinding process. It includes the hammering, abrasion, and extraction process for machining on RUM.

As per the work reported yet, Pei et al.^11^ is the first who one started the research on RUM of ceramic material. The RUM process can be employed for various operations such as drilling, grinding, and face milling of ceramics at different process parameters. After that, it is accelerated by Hu et al.^12^ for zirconia ceramic. It is found that the maximum material removal rate (MRR) is achieved at a power rating of 40–70%. In 2005, Li et al.^13^ employed RUM for machining two different ceramic composites. Zeng et al.^14^compared ultrasonic machining with RUM for ceramic materials. It is observed that RUM provided better MRR than ultrasonic machining. Zhang et al.^15^employed RUM for machining operations on K9 glass. It is found that the rotational speed has no significant impact on productivity. Lv et al.^16^ used RUM for BK7 glass. It is observed that the subsurface damage in RUM differentiated as grinding, chipping, and cracking on the glass. Besides this, numerous research work is also carried on titanium alloys by employing RUM^17^. Furthermore, few research works are carried out on the use of optimization techniques. Cong et al.^18^ created an experimental design technique for predicting the cutting force of CFRP material.in rotary ultrasonic machining. The developed model can predict the cutting force based on input variables i.e. tool amplitude, tool rotational speed, feed rate, abrasive mesh size and abrasive particle’s concentration. Lui et al.^19^ investigated the microchipping at the outside of the hole during the drilling process on RUM. The experiments are designed as per the response surface methodology using desirability approach. Teimouri et al.^20^ conducted the experiments with ultrasonic machine over titanium alloy grade- I using two different tools; high carbon steel (HSC) with hardness of 56 HRC and the titanium (Ti) alloy with hardness of 42 HRC. With the regression model multi objective optimization technique was employed and compare the data with other algorithms. The results indicated that the ICA outperforms the other algorithms in both cases of execution time and values of objective function at global optima. In the present study, a mechanistic model of workpiece deflection applicable to both conventional and UAD has been developed.

It is revealed from the literature review that the previous reported works focussed on RUM of ceramics, titanium, and glass. there has been only few research study reported on RUM of nickel based super alloys material. Nickel based alloys has wide application in the fabrication of jet engine and nuclear reactor structure^21–23^.The use of response surface methodology (RSM) with a view to design the experiments along with the assessment of parameters’ influence on process responses has also not been carried out so far. The parameter termed as ‘‘abrasive grit size” of tool has been omitted throughout many the investigation performed in RUM of numerous work materials. The variable ‘‘ultrasonic power’’ has been investigated at very low level (30–40%) in the past research studies. Thus, there is a need to expose the machining of Inconel 718 at higher power levels. In the contemplation of the above discussion, this article has been targeted to explore the impact of several process factors such as feed rate, spindle speed, ultrasonic power, abrasive grit size on machining characteristics, that is, MR, and Ra in RUM of Inconel 718 by employing RSM in the form of central composite design (CCD). A statistical tool ‘analysis of variance’ (ANOVA) is also utilized to check the viability of the statistical model. The mathematical model developed through this approach will be helpful in industrial revelation. The optimization of machining characteristics, that is, MR and Ra on machined surface with PSO (Particle swarm optimization) has also never been attempted earlier in reported studies on RUM. The concurrent optimization of both the machining responses will further make the method’s applicability more meaningful while settling real-life industrial problems. Multi-response optimization has been attempted to optimize MR and Ra simultaneously using MOPSO approach. Scanning electron microscopy (SEM) analysis of machined samples has been analysed and presented.

## Experimentation and methodology

### Workpiece and tool

**Figure 4:**
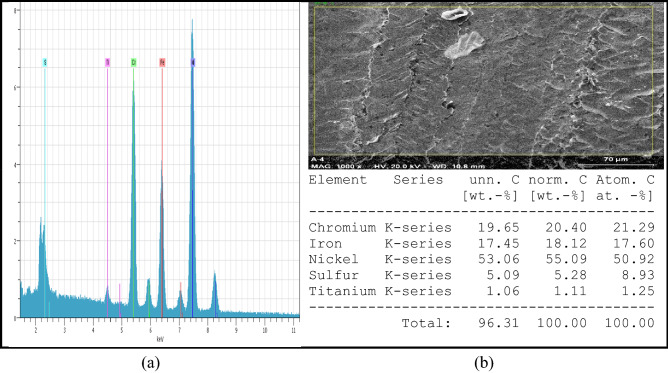
EDS analysis of Inconel 718.

**Figure 5:**
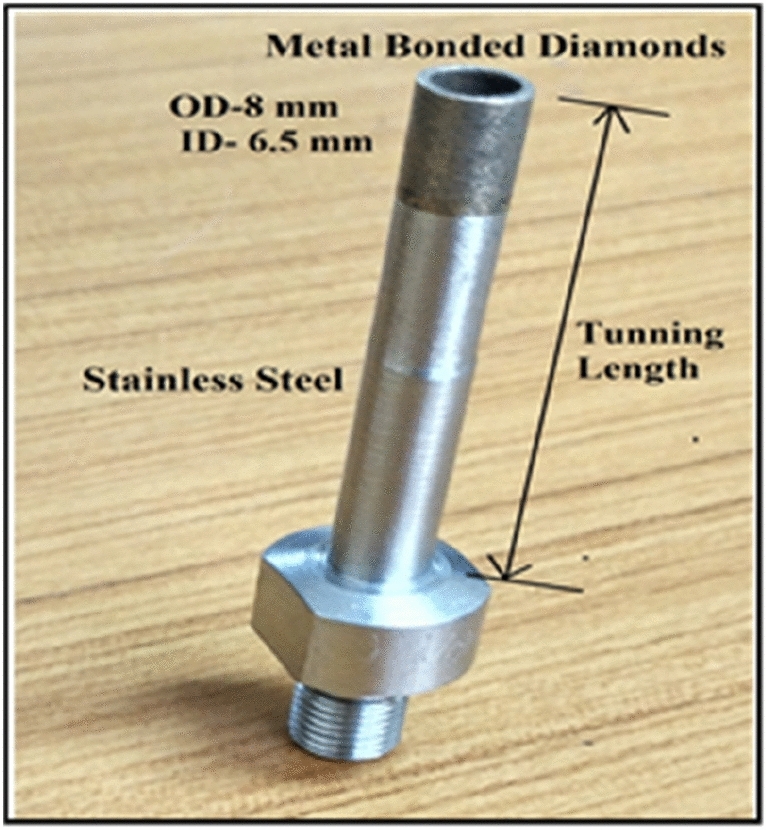
Photographic view of fabricated metal bonded diamond core drill picture.

In this current research, the work material Inconel 718 is selected for trials. The dimension of the square sheet is 50 × 50 × 5 mm. The properties of the material are depicted in Table 1. An EDS test is carried out before machining to ensure the quality of the workpiece. Figure 4a and b shows the results of EDS test. For the drilling of workpiece, a metal bonded diamond core drill tool is used for Inconel 718. Figure 5 shows the pictorial view of the diamond core drill tool. The outside (OD) and inside (ID) diameters of the diamond core drill tool are selected as 8 mm and 6.5 mm, respectively.

**Table 1:** Chemical and mechanical properties of Inconel 718.

Chemical composition (by weight %) of Inconel 718												
Element	Ni	Cb	Cr	Nb	Mn	C	Co	Al	Si	Ti	Mo	Fe
Weight (%)	50–55	4.75–5.5	17–21	5.7	0.35 max	0.08 max	0.2	0.2–00.8	0.35 max	0.65–1.15	2.8–3.3	Balance
Mechanical properties												
Yield strength	1034 MPa											
Ultimate strength	1242 MPa											
Hardness	97 HRB											
Specific Gravity	8.19 g/cm^3^

### Experimental setup and methodology

**Figure 6:**
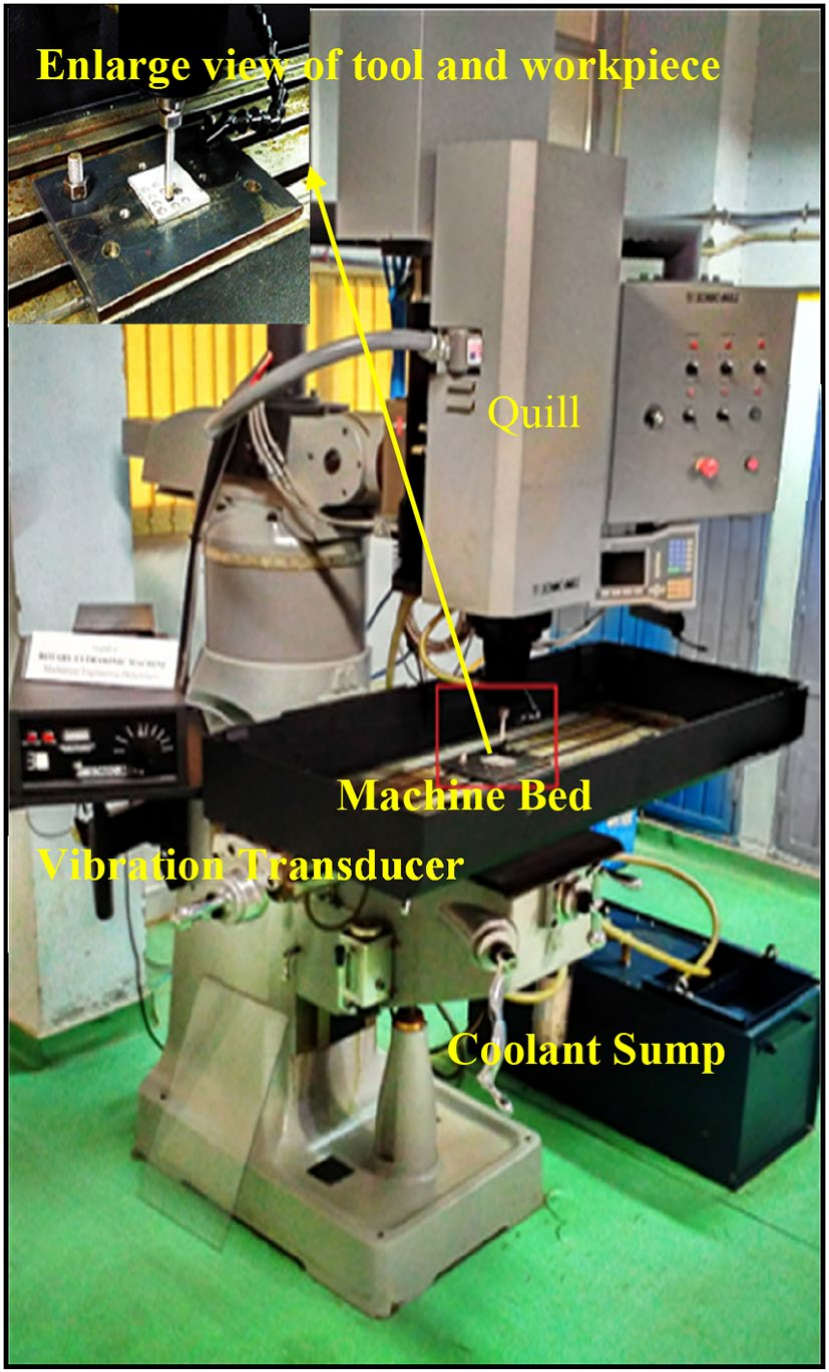
Experimental Setup of RUM.

In present research work, RUM (Sonic-Mill Series 10-Sonic-Mill, Albuquerque, NM, United States) is used for drilling operations for Inconel 718. Figure 6 depicts the photographic view of experimental setup. For finding the optimum results, the various trials are executed at different level of the process parameters i.e. tool rotation, feed rate, power rating, and abrasive size of the diamond. Table 2 depicts the different values of input machining parameters for present study. Besides this, the other process parameters like tool diameter 8 mm, the frequency of vibration 21 kHz, the amplitude of vibration 25.3–25.8 µm, and coolant pressure 300 kPa are kept constant. In addition, a diluted water-soluble coolant cum cutting oil (*Mobilmet S-122, Mobil Oil Corporation, Fairfax, VA, United States*) having oil to water ratio of 1:20 is employed during cutting operation in order to remove the heat and debris during the process.

**Table 2:** Different levels of paremeters.

Sr. no	Symbol	Input factors	Levels					Units
			− 2	− 1	0	+ 1	+ 2	
1	A	Tool rotation	4200	4600	5000	5400	5800	RPM
2	B	Feed rate	0.01	0.0125	0.0150	0.0175	0.02	mm/sec
3	C	Power rating	55	60	65	70	75	%
4	D	Diamond abrasive size	80	100	120	140	160	mesh

The experiments are designed as per central composite design (*rotatable design*)*.* As per the design, a total 21 experiments are carried out. Table 3 represents the experiments design for present research work. The tests are performed with a two replication. The average value of the observations is given in Table 4.

**Table 3:** Experimental design as per CCD.

Std	Run	Tool rotation (A)	Feed rate (B)	Ultrasonic power (C)	Diamond abrasive size (D)
		Rpm	mm/sec	%	Mesh
15	1	5000	0.01	65	120
18	2	5000	0.015	65	120
6	3	5000	0.02	65	120
21	4	4600	0.0125	60	100
14	5	5800	0.015	65	120
11	6	5000	0.015	65	80
2	7	5000	0.015	55	120
8	8	5000	0.015	75	120
10	9	4600	0.0175	70	140
17	10	5000	0.015	65	120
5	11	5000	0.015	65	160
19	12	5400	0.0125	60	140
3	13	5400	0.0175	60	100
16	14	4600	0.0175	60	140
9	15	5400	0.0175	70	100
20	16	5000	0.015	65	120
12	17	5400	0.0125	70	140
1	18	5000	0.015	65	120
7	19	4600	0.0125	70	100
4	20	4200	0.015	65	120
13	21	5000	0.015	65	120

**Table 4:** Design Matrix and their results.

Std	Run	Tool Rotation (rpm)	Feed Rate (mm/sec)	Ultrasonic Power (%)	Diamond Abrasive Size (Mesh)	*MR* (mm^3^/sec)	*R*_*a*_ (µm)
15	1	5000	0.01	65	120	0.4382	0.711
18	2	5000	0.015	65	120	0.7047	1.016
6	3	5000	0.02	65	120	1.0020	1.398
21	4	4600	0.0125	60	100	0.6077	1.033
14	5	5800	0.015	65	120	0.7045	0.912
11	6	5000	0.015	65	80	0.7311	1.233
2	7	5000	0.015	55	120	0.7110	1.019
8	8	5000	0.015	75	120	0.6890	1.005
10	9	4600	0.0175	70	140	0.8226	1.196
17	10	5000	0.015	65	120	0.7104	1.041
5	11	5000	0.015	65	160	0.7568	1.131
19	12	5400	0.0125	60	140	0.5434	0.603
3	13	5400	0.0175	60	100	0.8795	1.159
16	14	4600	0.0175	60	140	0.8078	1.311
9	15	5400	0.0175	70	100	0.8912	1.136
20	16	5000	0.015	65	120	0.7203	1.001
12	17	5400	0.0125	70	140	0.5455	0.902
1	18	5000	0.015	65	120	0.7014	0.999
7	19	4600	0.0125	70	100	0.6207	0.98
4	20	4200	0.015	65	120	0.7102	1.161
13	21	5000	0.015	65	120	0.7114	0.996

### Machining rate (MR) and surface roughness (R_a_)

In current study, machining rate (MR) and surface roughness (R_a_) are considered as response parameters. The machining rate is calculated from the weight measurement method. In this method, an electronic weighing machine (± 0.0002 g) is utilized for calculating the weight of the workpiece before and after each experiment. The Eq. (1) is used for calculating the MR. The volume is calculated by multiplying the density to the mass. The surface roughness of sample is computed by using roughness tester (*Make: Surfcom, Flex*)

1 $$Machining\, rate = \frac{Volume \;of\; material\; removed \;from\; workpiece}{{time\; of\; machining}} .$$

## Results and discussions

Table 4 depicts the outcomes of the current study. It represents the average values of MR and R_a_ of two experiments for each input value. It is observed that maximum MR is obtained in experiment run 1 whereas minimum roughness is obtained in experiment run 12. Three criteria are used such as lack-of-fit test, the sequential model sum of squares, and model overview statistics. For better results, a backward elimination process is used to eliminate the insignificant terms in the models. This elimination process enhances the adequacy of the model by removing the non-significant terms from the quadratic model to preserve the model hierarchy.

### Analysis of MR and R_a_

Tables 5 and 6 shows the values of the results after the backward elimination process. It is clear from Tables 5 and 6 that all the input parameters are important. Besides this, F values and P values also tell about the adequacy of the model. This model's F Table -value is evaluated by splitting the average square value of the model into the average square residual values. The F-value defines the relation between model variance and the residual variance. If variance values are almost identical, the fraction is almost equal to 1 and the model does not have an important impact on performance. The obtained F value of the model for MR and Ra is 472.61 and 47.598, respectively and for both MR and Ra the P-value are less than 0.05. Tables 5 and 6 shows that the model obtained for MR and Ra significant^24^.

**Table 5:** ANOVA for response surface of *MR.*

Source	SS	df	Mean Sq	F value	*p* value	Prob > F
Model	0.30995	8	0.038743	472.361	< 0.0001	Significant
A-Tool rotation	0.00001	1	0.000007	0.08188	0.0096	
B-Feed rate	0.29987	1	0.299870	3656.02	< 0.0001	
C-Ultrasonic power	0.00003	1	0.000029	0.35749	0.04610	
D-Diamond abrasive grit size	0.00033	1	0.000330	4.02447	0.0379	
AB	0.00526	1	0.005261	64.1453	< 0.0001	
BC	0.00039	1	0.000392	4.87433	0.0486	
B^2^	0.00043	1	0.000425	5.18258	0.0419	
D^2^	0.00205	1	0.002049	24.9827	0.0003	
Residual	0.00098	12	0.000082			
Lack of fit	0.00079	8	0.000099	2.0791466	0.2502	Not significant
Pure Error	0.00019	4	0.000048			
Cor Total	0.31093	20				
Std. Dev	0.00828		R-Sq		0.987569	
Mean	0.70468		Adj R-Sq		0.985581	
C.V. %	1.176223		Pred R-Sq		0.977345	
PRESS	0.003935		Adeq Prec		95.73918

**Table 6:** ANOVA for response surface of *R*_*a*_*.*

Source	SS	df	Mean Sq	F value	*p* value	Prob > F
Model	0.3819	8	0.04774	47.598	< 0.0001	Significant
A-Tool rotation	0.0132	1	0.01321	13.173	0.0035	
B-Feed rate	0.2763	1	0.27635	275.499	< 0.0001	
C-Ultrasonic power	0.0004	1	0.00046	0.4662	0.03077	
D-Diamond abrasive grit size	0.0094	1	0.00945	9.4294	0.0097	
AC	0.0096	1	0.00963	9.6044	0.0092	
BC	0.0047	1	0.0047	3.3229	0.0485	
BD	0.0051	1	0.00514	5.1307	0.0428	
D^2^	0.0227	1	0.02277	22.705	0.0005	
Residual	0.0120	12	0.00100			
Lack of fit	0.0098	8	0.00123	2.3096	0.2183	not significant
Pure Error	0.0021	4	0.00053			
Cor total	0.3940	20				
Std. Dev	0.03167		R-Sq		0.96944	
Mean	0.82890		Adj R-Sq		0.94908	
C.V. %	3.82090		Pred R-Sq		0.85564	
PRESS	0.05687		Adeq Prec		25.8174	

The R^2^ is called the determination coefficient which tells the degree of closeness between experimental value and predicted value. The percentage of closeness to the 1 showed the good experimental value against the predicted value. In the present work, the obtained R^2^ value for MR and R_a_ came out to be 98.7% and 96.9% respectively as shown in Tables 5 and 6. Some more properties such as adjusted R^2^, predicted R^2^ adequate precision also plays a good role for the adequacy of the model. It shows healthy agreement between experimental value and predicted value. “Adequate precision” signifying the signal-to-noise ratio (S/N). In general, the value greater than 4 is acceptable^25^. In both ANOVA Tables 5 and 6 not only individual parameters but also in an interactive way it influences response parameters i.e., MR and Ra.

**Figure 7:**
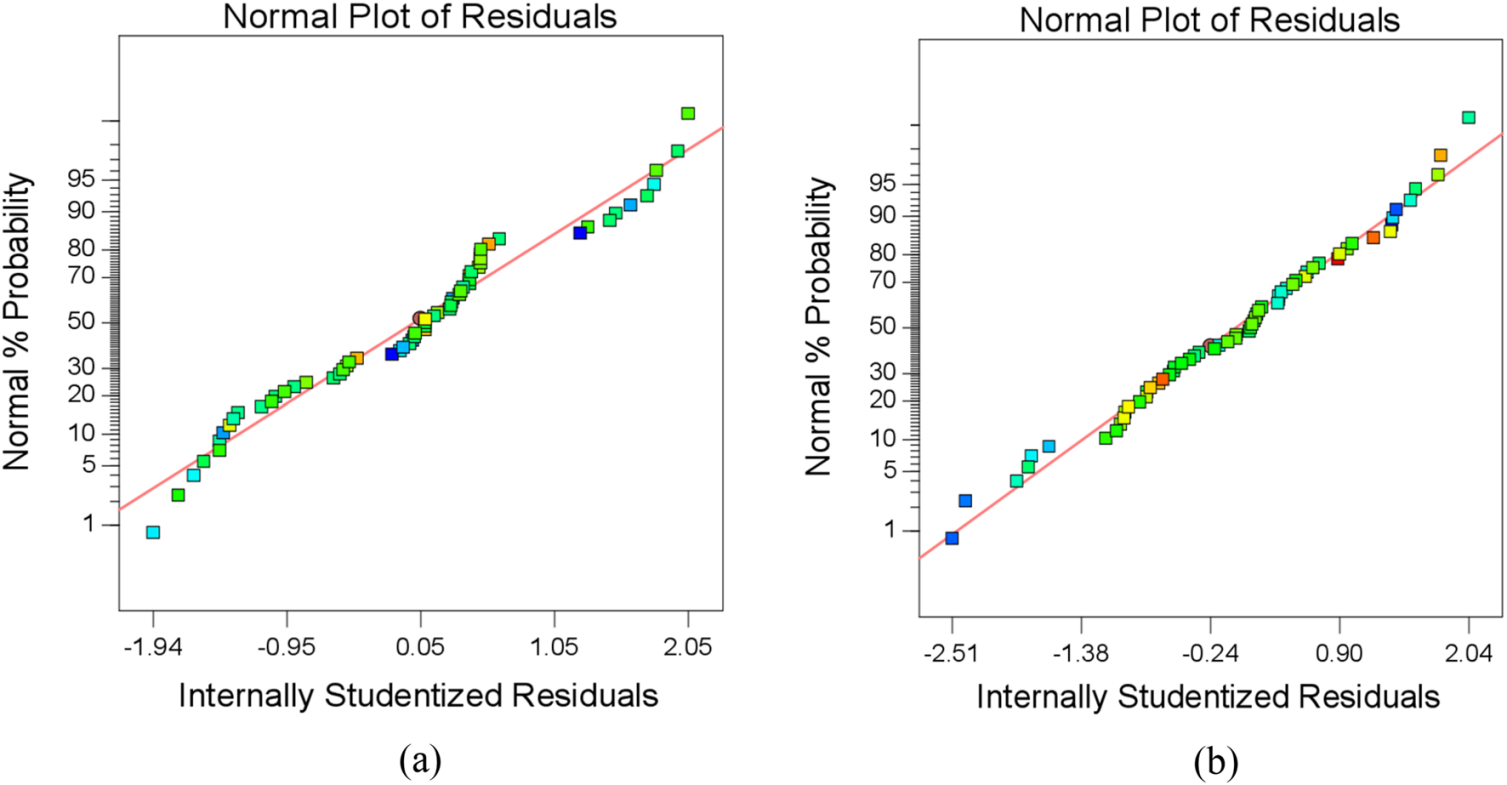
Residuals plots (**a**) MR and (**b**) R_a_.

**Figure 8:**
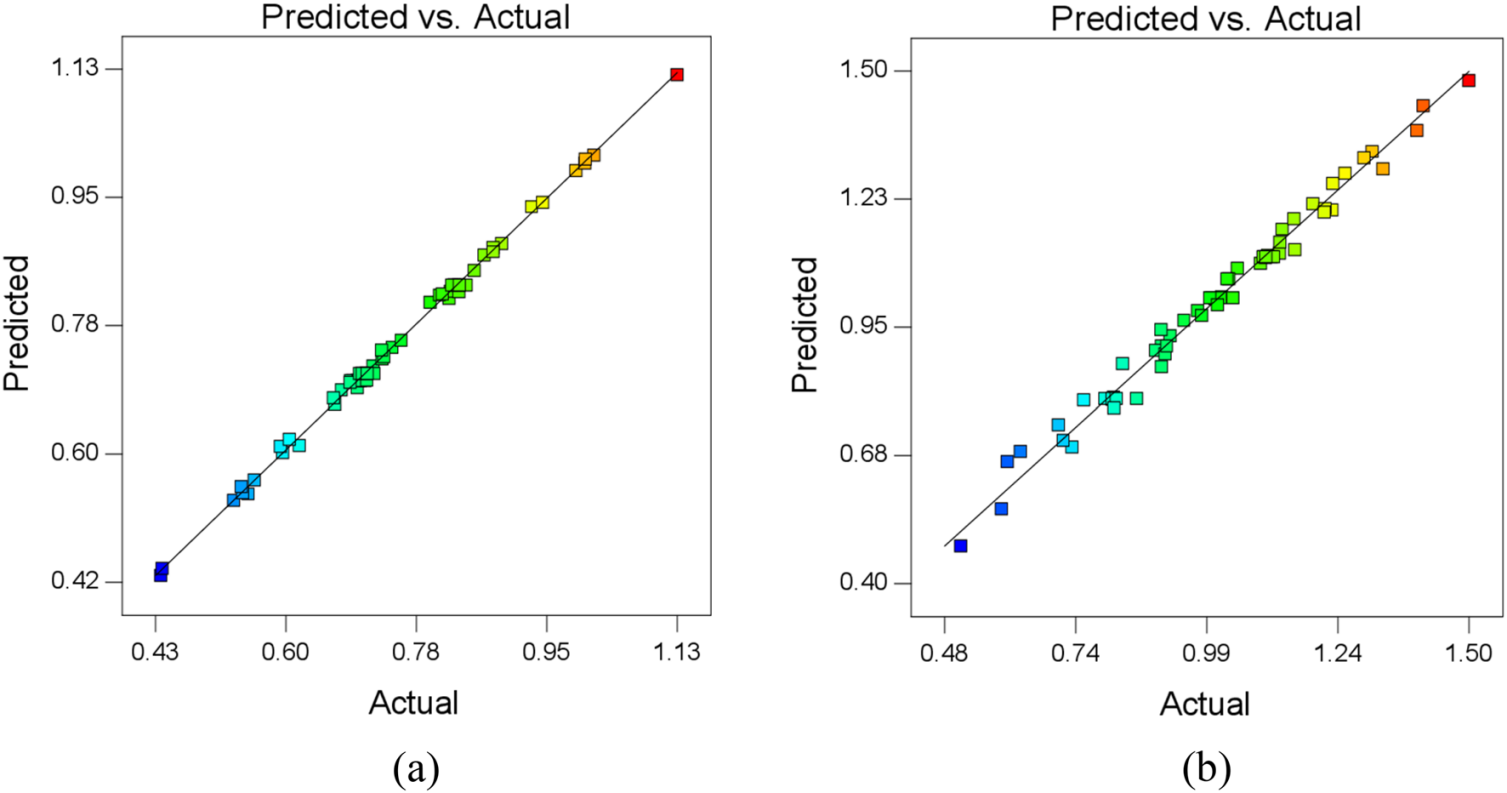
Predicted vs actual (**a**) MR and (**b**) R_a_.

Figure 7 represents the standard residual probability curve which shows that residues are inside ± 3 limits and that they are fixed by the MR and Ra straight lines. Figure 8 demonstrates that the estimated model values are true to MR and Ra experimental values. This reveals that ANOVA table findings are reliable. Equation (2) and (3) represents the regression model for MR and R_a_ respectively.

2 $$\begin{aligned} R = & + 3.52990 - 0.000542 \times Tool\, Rotation - 179.577 \times Feed \,Rate - 0.00812 \times Ultrasonic\, Power \\ & - 0.00488 \times Diamond\, Abrasive \,Grit \,Size \\ & + 0.0362 \times Tool \,Rotation \times Feed \,Rate + 0.523 \times Feed\, Rate \times Ultrasonic\, Power \\ & + 632.769 \times Feed\,Rate^{2} + 0.0000217 \times Diamond \,Abrasive\, Grit Size^{2} \\ \end{aligned}$$

3 $$\begin{aligned} R_{a} & = 7.135 - 0.00122 \times Tool \,Rotation + 71.014 \times Feed\, Rate - 0.06371 \\ & \;\;\;\; \times Ultrasonic\, Power - 0.02914 \times Diamond \,Abrasive\, Grit \,Size \\ & \;\;\;\; + 0.00001735 \times Tool \,Rotation \times Ultrasonic\, Power - 1.608 \\ & \;\;\;\; \times Feed\, Rate \times Ultrasonic \,Power + 0.717 \times Feed\, Rate \times Diamond\, Abrasive\, Grit \,Size \\ & \;\;\;\; + 0.0000715 \times Diamond\, Abrasive\, Grit\, Size^{2} . \\ \end{aligned}$$

### Process parameter effect analysis

**Figure 9:**
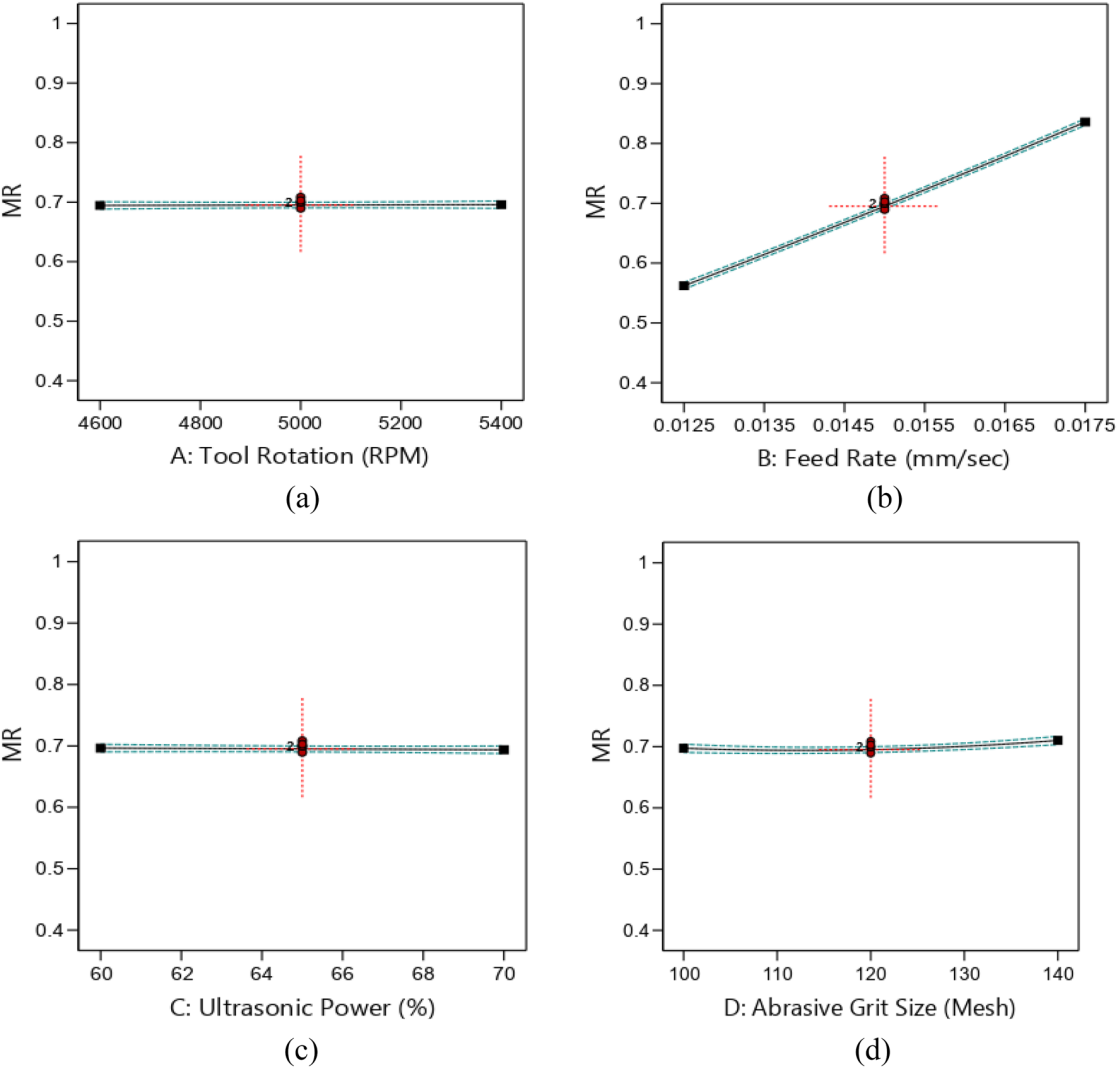
Effects of RUM parameters on MR (**a**) rotational speed, (**b**) feed rate, (**c**) ultrasonic power, (**d**) abrasive grit size.

Figure 9 depicts the impact of parameters i.e., rotational speed (rpm), feed rate (mm/sec), ultrasonic power rating (%) and abrasive grit size (mesh) on machining rate. It is observed that rotational speed does not significantly affect the MR as shown in Fig. 9a. Conversely, it is observed that the MR is significantly changed from 0.5512 to 0.8525 mm^3^/sec with change in the penetration rate from 0.0125 to 0.0175 mm/sec as depicted in Fig. 9b. It is attributed towards the deep grooving of abrasive particles at higher feed rate and resulted in higher MR. Figure 9c shows the impact of ultrasonic power on MR. It is visible that the *MR* is increased from 0.6965 to 0.7109 mm^3^/sec for an increase in power from 60 to 65%. Further increase in power upto 70%, *MR* decreases from 0.7109 to 0.6937 mm^3^/sec. The obtained results are consistent with the previous study of researchers^26,27^.

The effect of the size of the diamond on MR is shown in Fig. 9d. Abrasive grit size is inversely proportional to the mesh value of abrasive grit. It is observed that MR not significantly changed with change in the abrasive grit size. In addition, the curvature is observed in abrasive grit size effect on MR. It is an indication of the size of the grain of the diamond use in bonded tool enhance the machining rate. This is owing to deeper indentation of abrasive particles into the workpiece^28^.

**Figure 10:**
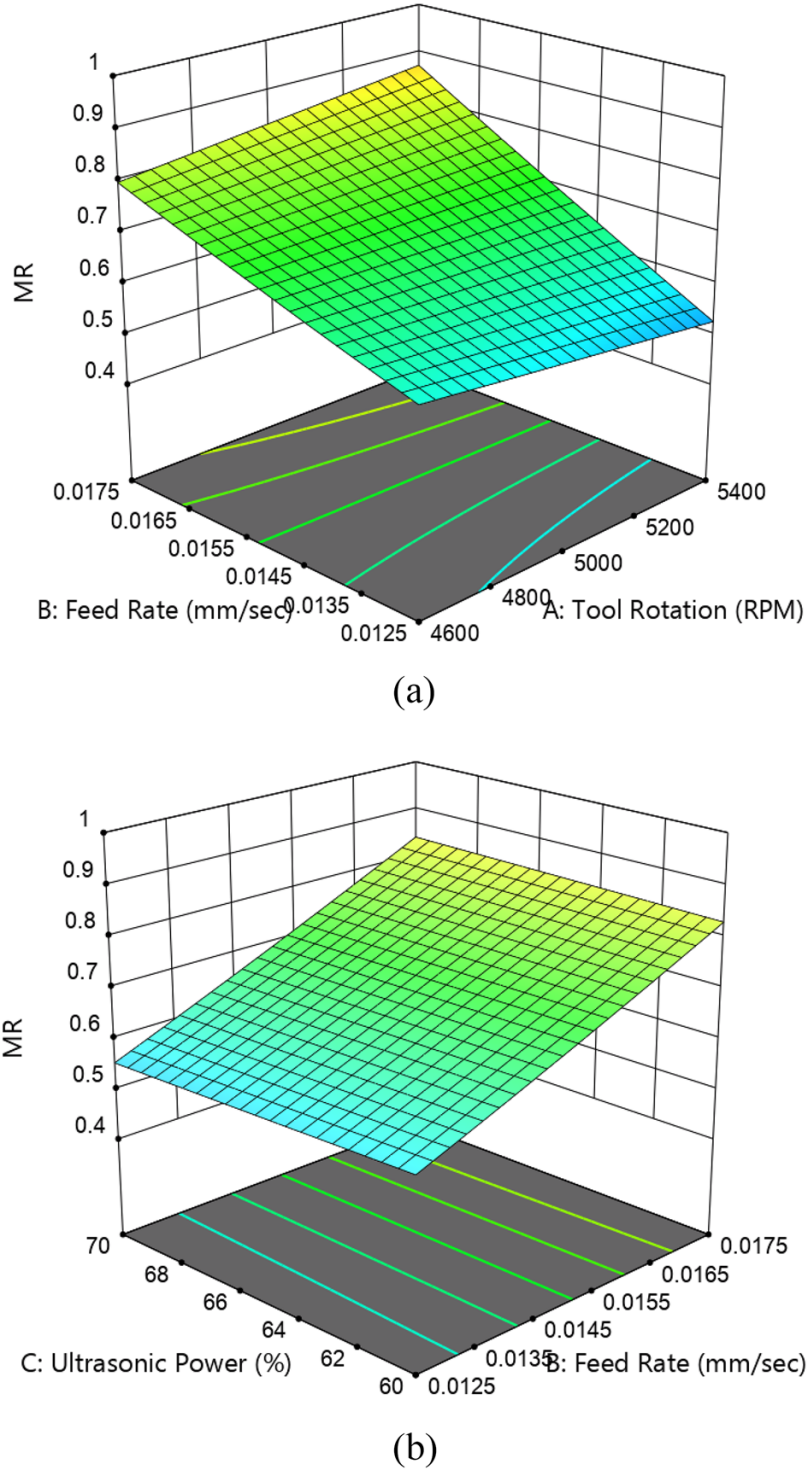
3D-contour plot of interaction effect (**a**) feed rate and tool rotation (**b**) feed rate and ultrasonic power on MR.

Figure 10a depicts the interaction effects on MR. It is verified from Eq. (2) that two interactions are found significant for MR. It is clearly visible that the maximum MR is obtained in a region where feed rate and tool rotation is high. It is attributed towards the increase in the contact length of diamond abrasive particles. Conversely, minimum *MR* is observed in a region where feed rate is low and tool rotational speed is higher. This is owing to lower point of contact between the tool and the workpiece. Interaction effect between the ultrasonic power and feed rate on MR is shown in Fig. 10b. It is observed that MR is achieved to be maximum in regions where feed rate and ultrasonic power is maximum. This happens due to increase in the vibration with increase in the ultrasonic force that eliminates swarf and debris efficiently from machining surfaces. On the other hand, minimum MR is obtained at lower penetration rate and ultrasonic power.

**Figure 11:**
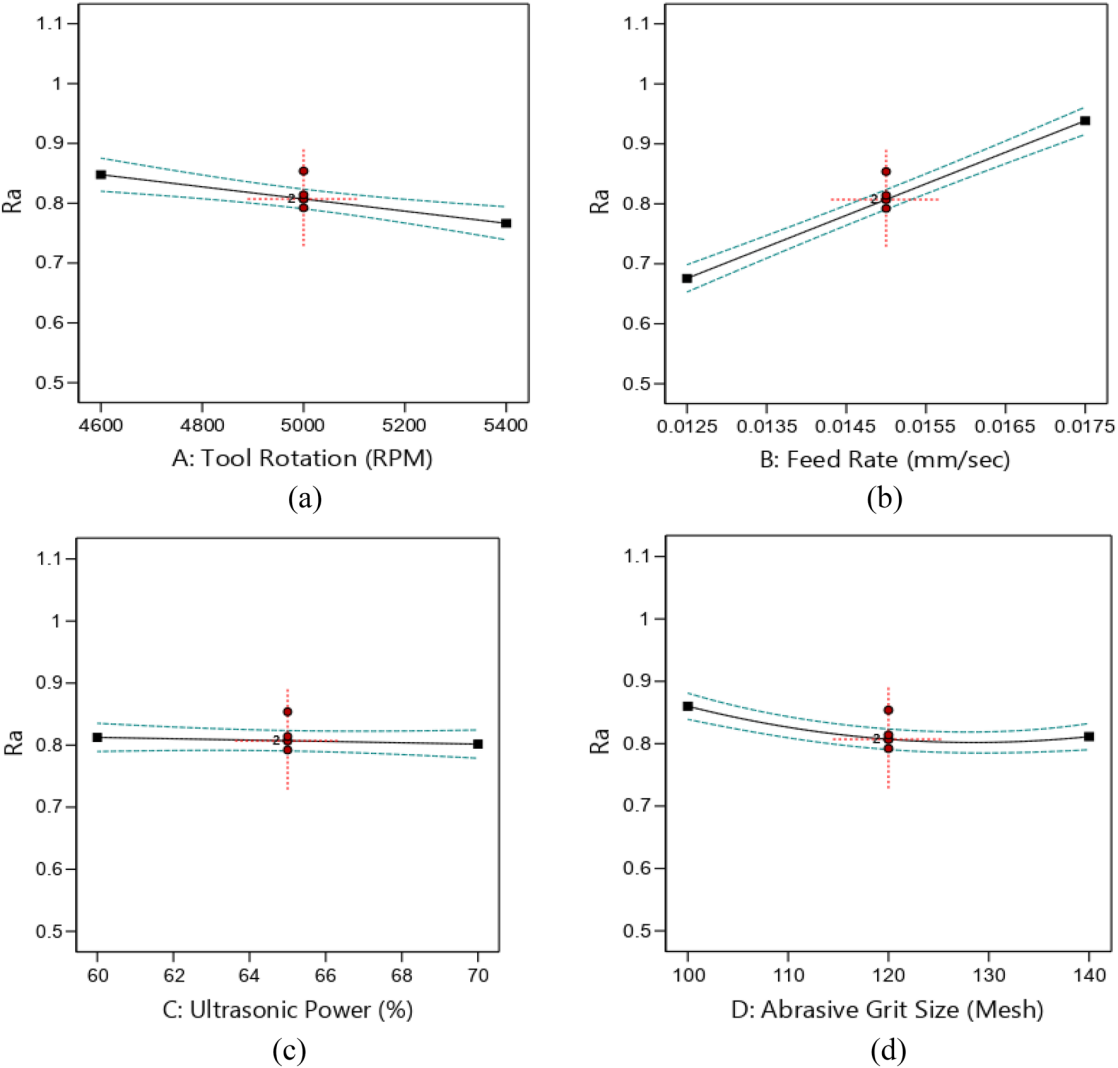
Effects of RUM parameters on *R*_*a*_ (**a**) tool rotational speed, (**b**) feed rate, (**c**) ultrasonic power, (**d**) abrasive grit size.

Figure 11 illustrates the impact of process parameters on the machined surface. Figure 11a depicts the effect of tool rotational speed on R_a_. It is concluded that the R_a_ is decreased with increase in the tool rotational speed. It is attributed towards the enhancement in the grinding action per unit time of the tool with increase in the rotational speed. Another reason for better surface roughness is to reduce the development rate of micro-cracks on the surface^29^. Figure 11b shows the effect of feed rate on R_a_. It is observed that the R_a_ is increased steeply from 0.676 to 0.938 µm with an increase in penetration rate from 0.0125 to 0.0175 mm/sec. This increase is owing to the extension in the micro-cracks on the workpiece surface. The effect of ultrasonic power on R_a_ is shown in Fig. 11c. It is found that the *R*_*a*_ is decreased with increase in the ultrasonic power. This change is not found to be significant. Moreover, it is also observed that the amplitude difference has no effect on the *R*_*a*_. Figure 11d depicts that *R*_*a*_ is decreased from 0.861 to 0.807 µm with increase in grit size from 100 to 120 mesh size. Conversely, it is increased little from 0.807 to 0.811 µm as grit size increases from 120 to 140 mesh. This is owing to coarse abrasive grains that results in enhancement of fracturing rate. In addition, during the RUM process, the diamond particles continuously move in the hole cavity. The increased size of the granulate increases the frictional forces at the lateral interface and contributes to the surface damage incurred by this uniform lateral wear^30^.

**Figure 12:**
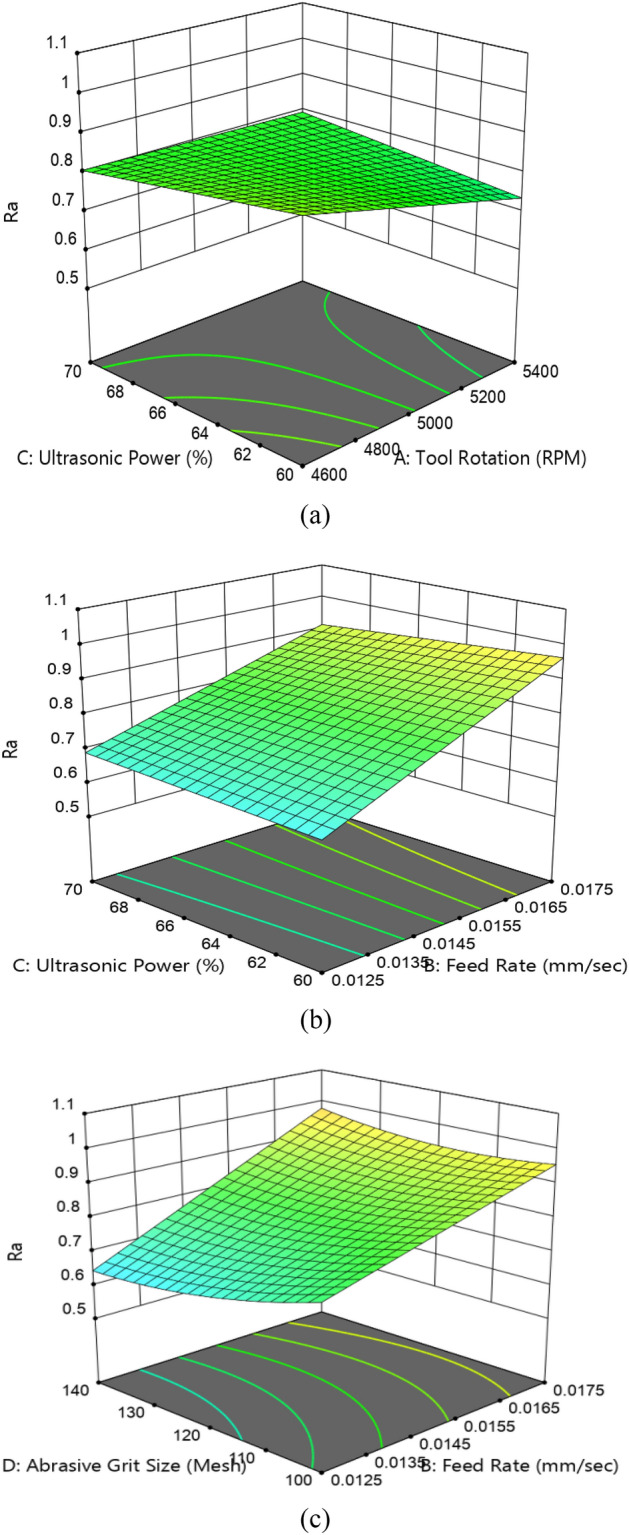
3D- interaction plot effect R_a_ (**a**) tool rotation and ultrasonic power, (**b**) feed rate and ultrasonic power, and (**c**) feed rate and diamond abrasive size.

Figures 12 illustrates the interaction effect on *R*_*a*_. It is verified from Eq. (3) that three interactions are found to be significant for *R*_*a.*_ Figure 12a shows the mix effect of ultrasonic power and tool rotational speed. The minimum value of *R*_*a*_ is obtained in an area where ultrasonic power is minimum and tool rotational speed is maximum. This is owing to an increase in the grinding pass of the tool that results in the fineness of machining surfaces. The interaction effect of feed rate and ultrasonic power on *R*_*a*_ is depicted in Fig. 12b. The minimum *R*_*a*_ of 0.665 µm is obtained in a region of low feed rate and low ultrasonic power. It is owing to lower abrasive diamond indentation depth on the workpiece. The maximum *R*_*a*_ of 0.965 is found in a region where feed rate is maximum and ultrasonic power is minimum. This is owing to higher indentation depth of abrasive particles on the workpiece surface. Figure 12c shows the effect of feed rate and abrasive grit size on Ra. It is clearly visible in Fig. 12c that the minimum value of *R*_*a*_ i.e., 0.762 µm is obtained for low feed rate and fine grit size (140 mesh). It is attributed towards lower indentation depth of diamond particles on the workpiece surface. The value of *R*_*a*_ is maximum at higher feed rate at all abrasive grit size. This is owing to a change in the grit size from coarse to fine i.e., 0.9083 µm^31^.

### Microstructural analysis of machined surface

**Figure 13:**
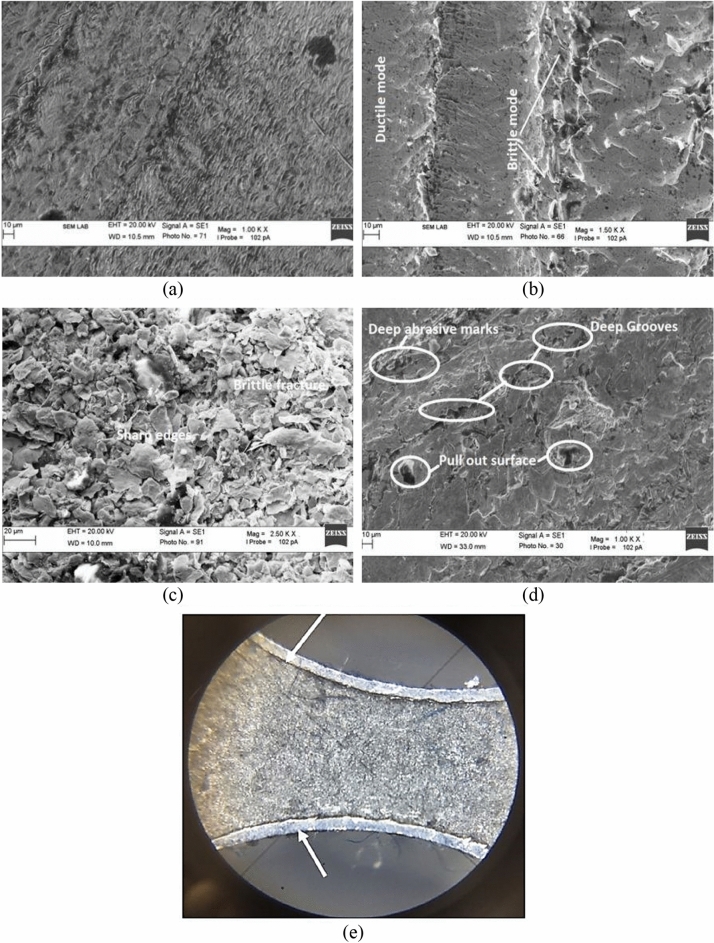
Micrograph of (**a**) Inconel 718 (prior to machining), (**b**) and (**c**) higher surface roughness specimen (**d**) minimum surface roughness specimen, (**e**) hole edge quality.

The SEM machine is utilized to study the surface of base material, maximum surface roughness specimen (experimental run 3, minimum surface roughness specimen (experimental run 12) as shown in Fig. 13. This is evident from Fig. 13a that the surface of the base material (Inconel 718) is uniform without any micro-cracks and grooves. Conversely, the machined surfaces consisted of microcracks and grooves on the surface. Figure 13b and c depicts the surfaces of maximum roughness surfaces. Two kinds of fracture are observed on the machined surface of maximum surface roughness i.e., ductile fracture and brittle fracture as shown in Fig. 13b. In addition, sharp edges, deep holes, and micro-cracks are also observed on the machined surface as shown in Fig. 13c. Due to high feed rate of the tool, the material removed from the surface in bigger chunk. Furthermore, sometimes depleted edges are also observed on the machine surface. It is an indication of brittle fracture that showed the promulgation of intergranular and trans-granular cracks. These types of surfaces are observed owing to vibration movement of the tool during the process. Figure 13d depicts the machined surface of minimum surface roughness. Small holes and deep abrasive marks are observed on the surface. Moreover, the edge quality of the machined workpiece is also analysed by using an optical microscope as shown in Fig. 13e. There is no crack and burr is observed on the drilled hole edge.

### Optimization through particle swarm optimization

The word “Optimization” means to make the best possible use of resources. In present research, a metaheuristic optimization technique i.e., particle swarm optimization (PSO) is also used to get the optimum values of process parameters of RUM for Inconel 718. According to best knowledge of the author, Kennedy and Eberthart^32^ introduced PSO in 2006. It is a stochastic algorithm that is capable of solving optimization problems with the evolutionary algorithm such as genetic algorithm, differential evolutionary, etc. This technique is also capable of producing the food searching behaviour of society such as a bird swarm or school of fish. Each member of the swarm in PSO is considered a particle. Each and every particle in the search space represents the potential solution. In addition, the information collected from the particles is sorted for getting the best particle in the swarm such as global best (gbest).

**Figure 14:**
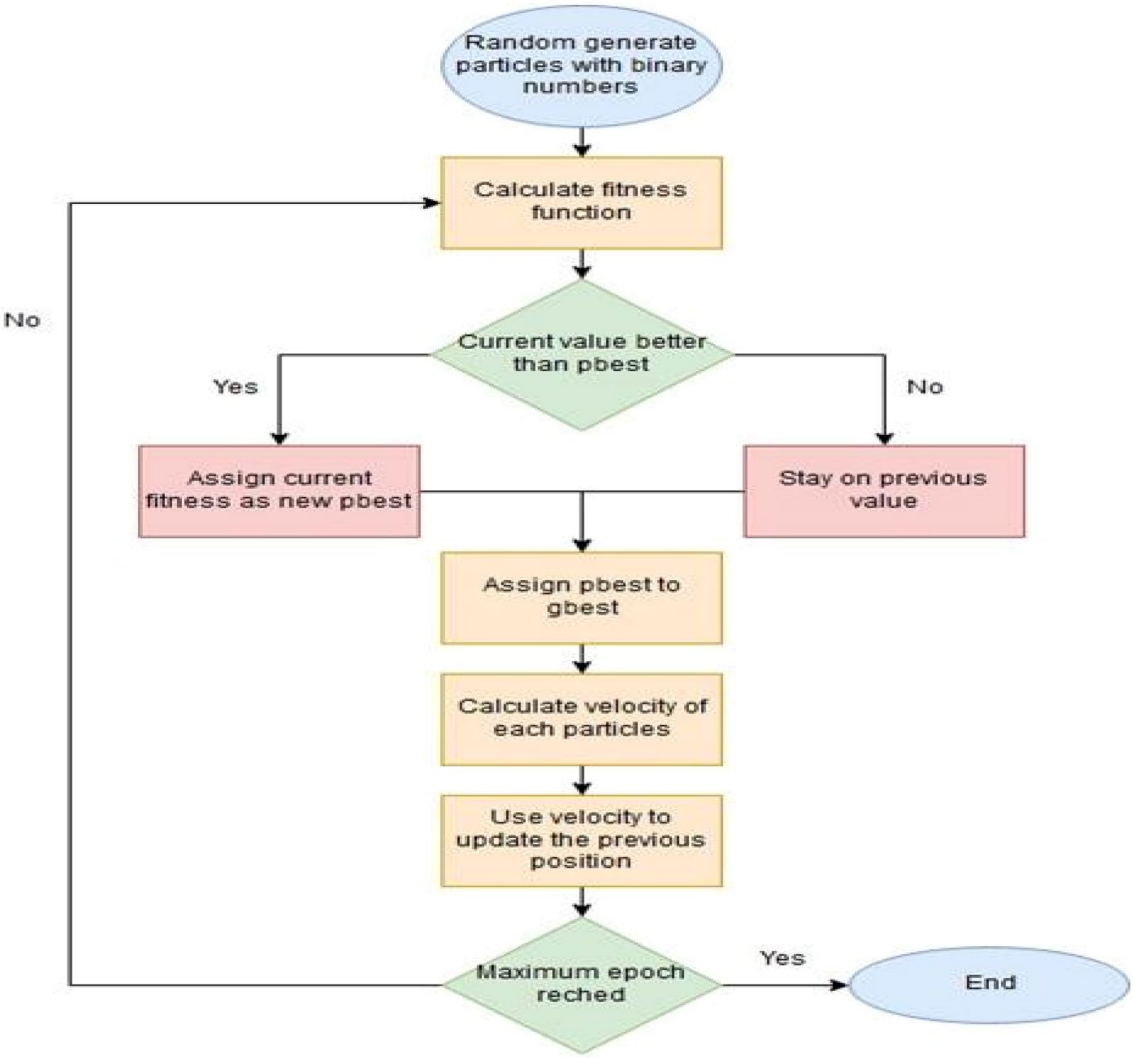
Flow chart of particle swarm optimization.

Moreover, each particle position is defined in terms of vectors such as position vector and velocity vector. The position and the velocity vector of the $i^{th}$ particle in the *d*-dimensional search space can be expressed as $x_{i} = \left( {x_{i1} , x_{i2} , ..., x_{id} } \right)$ and $v_{i} = \left( {v_{i1} , v_{i2} , ..., v_{id} } \right)$ respectively. The best location of each particle is dependent on the user defined fitness function i.e. $p_{i} = \left( {p_{i1} , p_{i2} , ..., p_{id} } \right)$, denoted as pbest and the fittest particle found in the complete set of swarm is $p_{g} = \left( {p_{g1} , p_{g2} , ..., p_{gd} } \right)$, denoted as gbest. The both values are corresponding to its best fitness values at time (t). Equation 4 and 5 are used for calculating the new positions and new velocity vectors for next fitness evaluation at time (t + 1).

4 $$x_{id} \left( {t + 1} \right) = x_{id} \left( t \right) + v_{id} \left( t \right)$$

5 $$v_{id} \left( {t + 1} \right) = w_{id} \left( t \right) + c_{1} rand_{1} \left( {p_{id} \left( t \right) - xid\left( t \right)} \right) + c_{2} rand_{2} \left( {p_{gd} \left( t \right) - x_{id} \left( t \right)} \right)$$

where $rand_{1}$ and $rand_{2}$ are the random values which fall between (0, 1), w is the factor inertia weight, and used to give the direction of previous velocities on the present particle velocity, $c_{1}$ is cognitive learning factor that shows the movement of particle toward its own success and $c_{2}$ defines the social learning factor which shows that a particle moves toward near its neighbour’s value. Some researcher has suggested the ranges of $c_{1}$ as (1.5 to 4) and $c_{2}$ as (2 to 2.5). Figure 14 represents the flow chart of PSO technique.

#### Coding of particles

The binary code is used to generate the particle in PSO. The binary format particle is decoded by using Eq. 6. The accuracy is given by Eq. 7.

6 $$X_{i} = X_{i}^{l} + \frac{{X_{i}^{U} - X_{i}^{L} }}{{2^{n} - 1}}S_{i}$$

where $X_{i}$: the decoded value of RUM parameters. $X^{L}$: is the lower limit of RUM parameters. $X^{U}$: the upper limit of RUM parameters. n: is the substring length (= 4). $S_{i}$ is the decoded value of the $i^{th }$ particle

7 $$Accuracy = \frac{{X^{U} - X^{L} }}{{X^{n} - 1}}.$$

#### Crowding distance

The crowding distance is a key concept for sorting the options into upward objective values. It is the mean of two adjacent solution values. Infinite crowding distance values are given to the boundary solutions that have the lowest and highest objective function values, so they are often chosen. For each objective function, this step is completed. A solution's final crowding distance value is determined by applying all the different crowding distance values to each objective function. The algorithm for crowding distance is listed below.
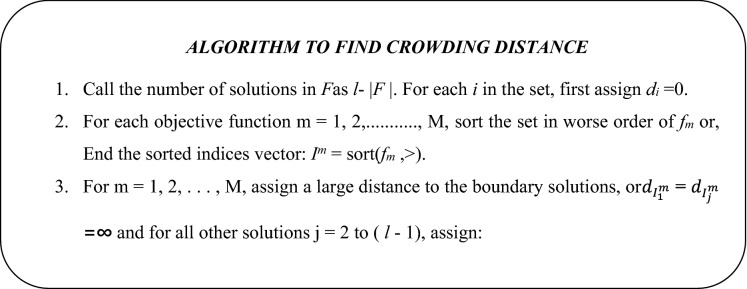


**Figure 15:**
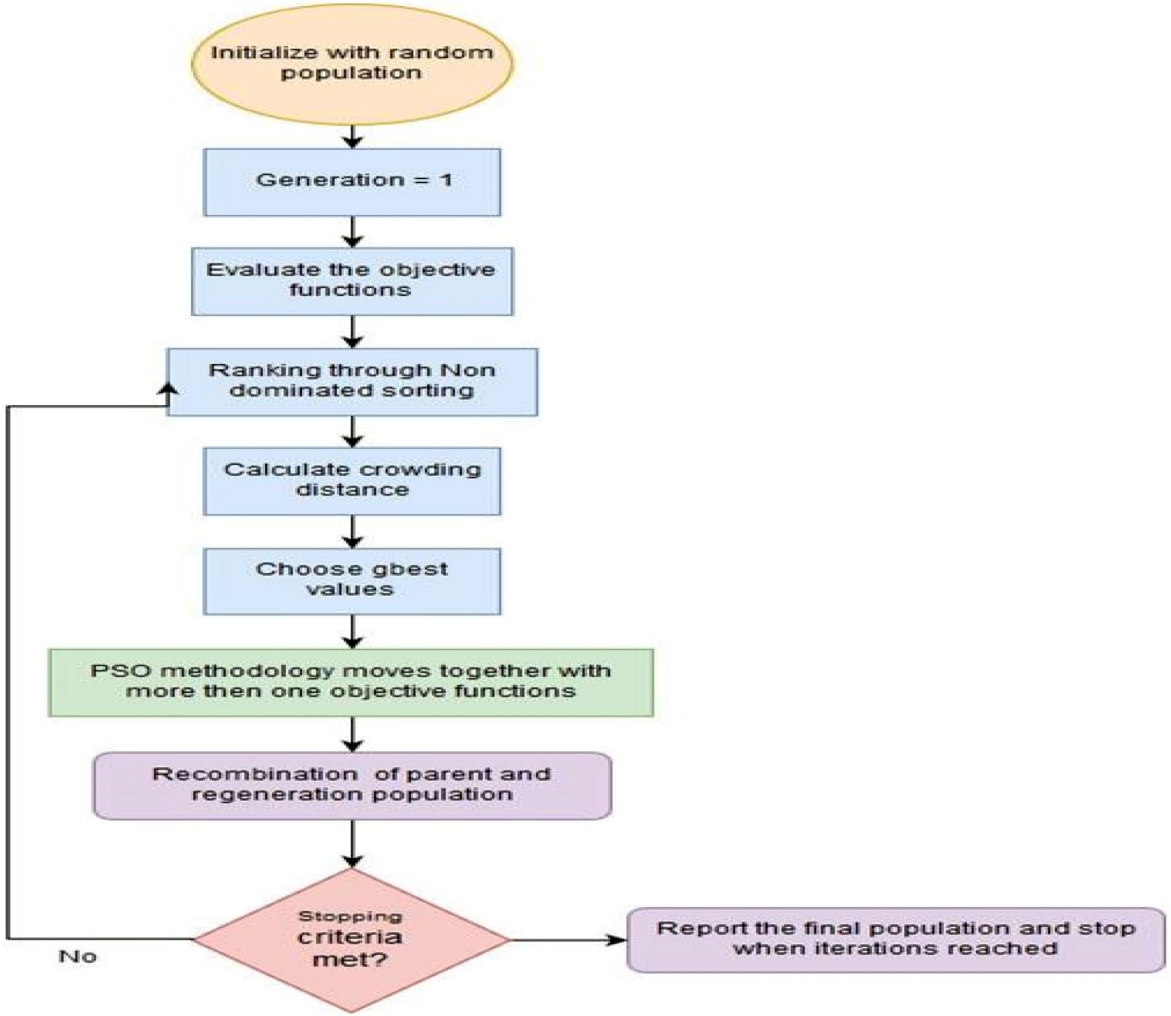
Flow chart of MOPSO.


Figure 15 represents the multi-objective PSO (MOPSO) flow chart. The algorithm for MPSO is listed below.

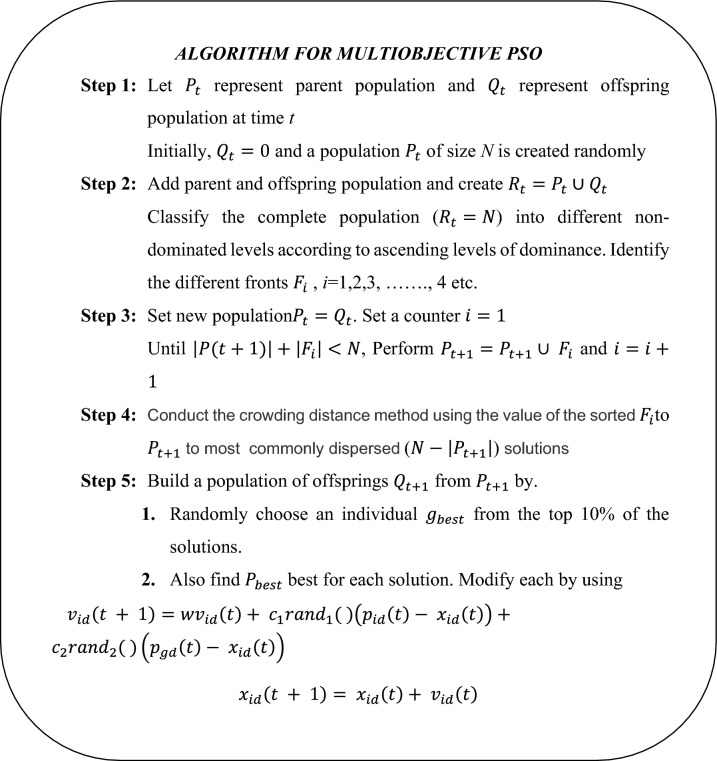
Where $rand_{1}$ and $rand_{2}$ are random numbers between 0 and 1. If the current position beyond the limits takes the upper or lower limits and its velocity is generated randomly. Finally perform the steps 2–5 until stopping criteria are met.

In the current research work, *MR* and *Ra* both responses are opposing in design. It means higher value of machining rates resulted in higher value of surface roughness. In order to achieve a higher machining rate with better surface finishing, optimal parameter’s values must be obtained. For finding the better value of the machining rate and surface roughness, single and multi-objective PSO is used. The lower and upper bound values of the parameters are used in algorithm so that the value should not go over bound. The values are given in Table 7.

**Table 7:** Lower and upper bound of RUM parameters.

	Tool rotation (rpm)	Feed rate (mm/sec)	Abrasive grit size (mesh)	Ultrasonic power rating (%)
Lower bound	4600	0.01	80	55
Upper bound	5800	0.02	140	75

#### Maximization of MR

**Figure 16:**
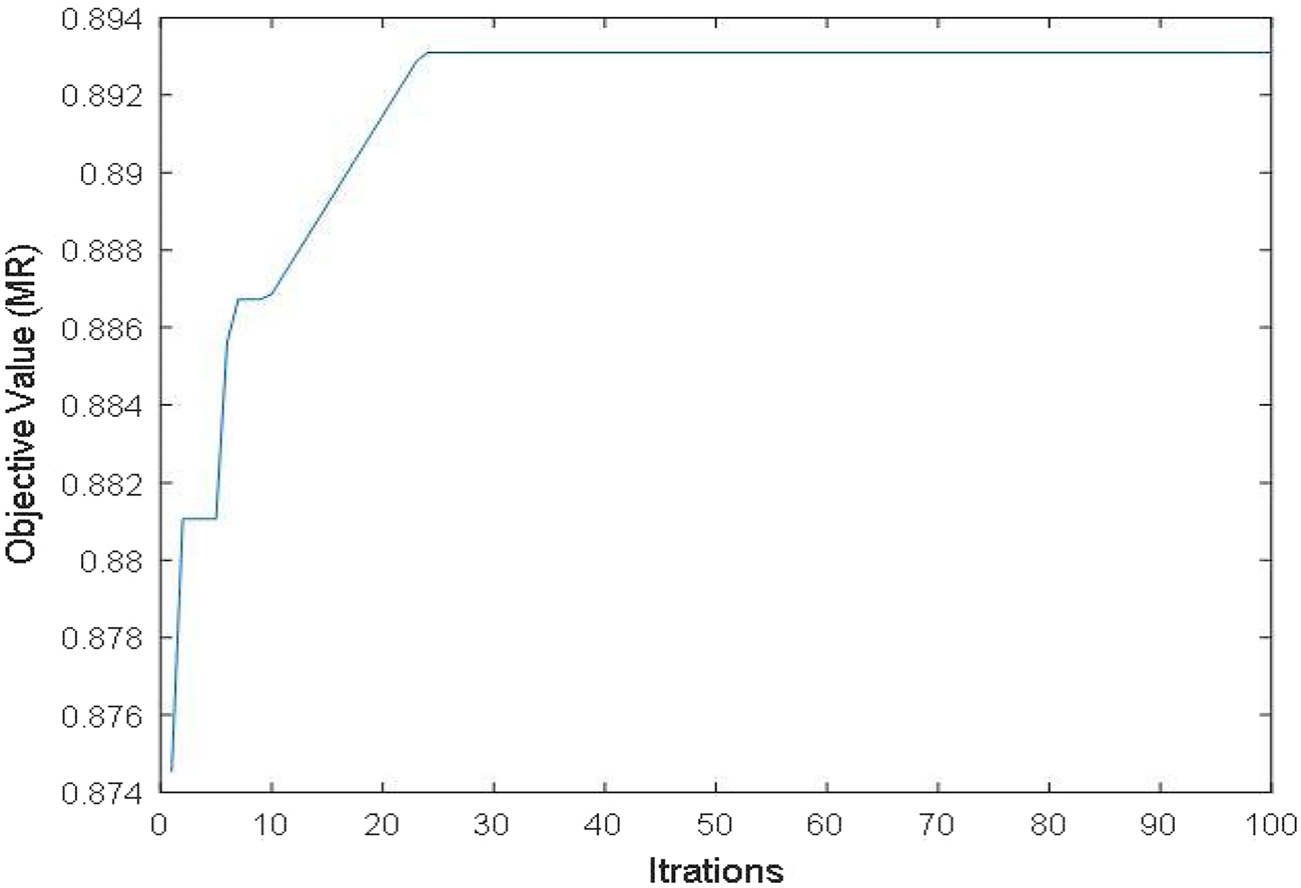
Iteration plot for MR.

The developed empirical model [Eq. (2)] is utilized for implementing the PSO technique. Figure 16 depicts the values of MR with each iteration after employing the PSO technique on empirical models. After successive iterations, PSO gives the maximum value of MR (0.8931 mm^3^/sec) at parameters combination of Tool Rotation-5400 rpm; Feed Rate-0.0175 mm/sec; Ultrasonic Power-70%; Diamond Abrasive Grit Size- 140 mesh depicted in Table 8.

**Table 8:** Optimum values of RUM conditions for *MR.*

RUM Parameters				Predicted value from PSO	Confirmation through experiments
Tool rotation	Feed rate	Ultrasonic power	Diamond abrasive grit size	MR	MR
5400 rpm	0.0175 mm/sec	70%	140 mesh	0.8931 mm^3^/sec	0.8625 mm^3^/sec

#### Minimization of* R*_*a*_

**Figure 17:**
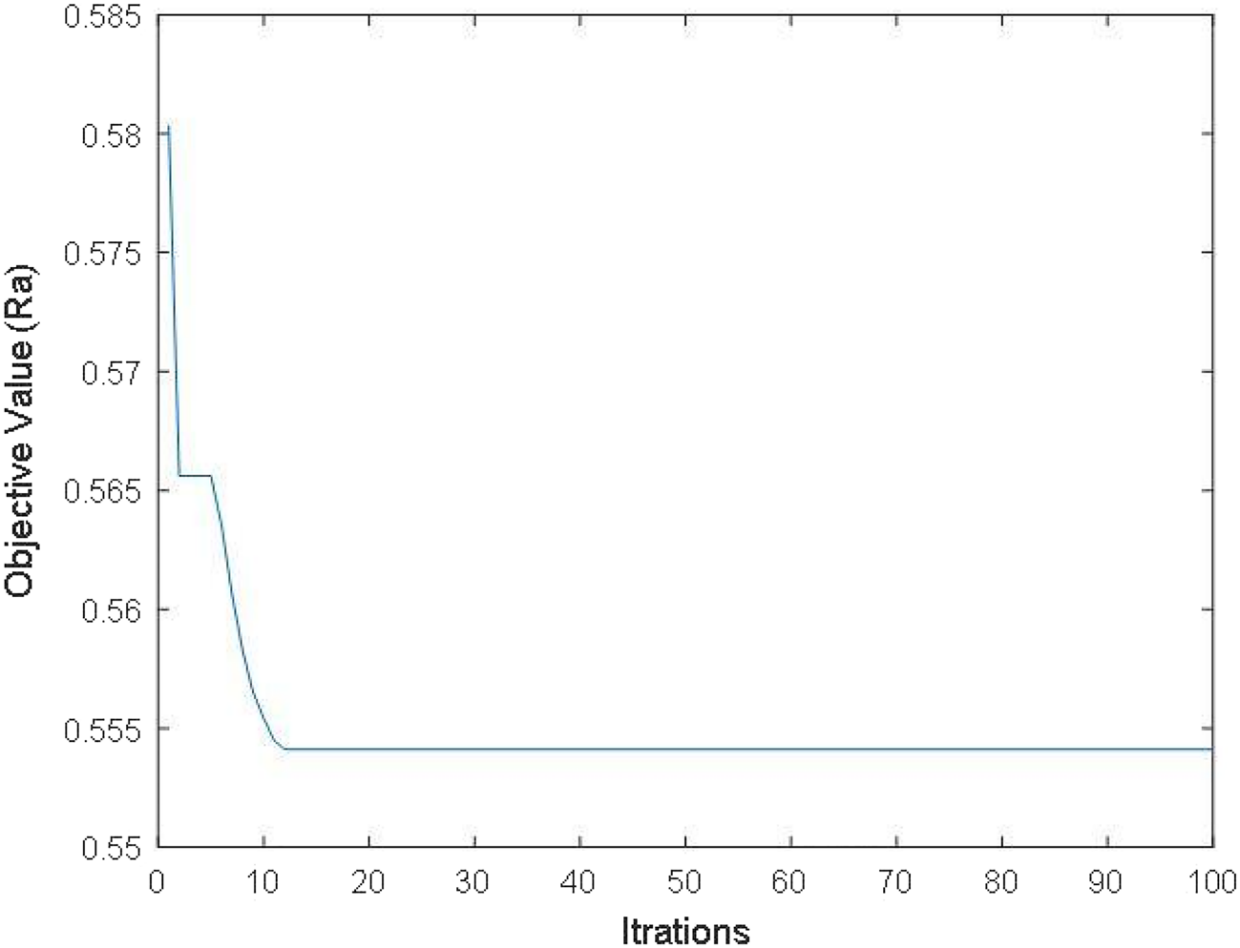
Iterations plot for Ra.

To predict lower value of Ra, the empirical model (Eq. 3) is used in PSO. The predicted values of *R*_*a*_ for each iteration during PSO technique is depicted in Fig. 17. After successive iterations, PSO gives the minimum value of Ra (0.554 µm) at parameters combination of Tool Rotation- 5400 rpm; Feed Rate-0.0125 mm/sec; Ultrasonic Power-60%; Diamond Abrasive Grit Size- 140 mesh depicted in Table 9.

**Table 9:** Optimum values of RUM conditions for *R*_*a*_*.*

RUM Parameters				Predicted value from PSO	Confirmation through experiments
Tool rotation	Feed rate	Ultrasonic power	Diamond abrasive grit size	*R*_*a*_	*R*_*a*_
5400 rpm	0.0125 mm/sec	60%	140 mesh	0.554 µm	0.572 µm

For the validating these results confirmation tests are conducted on RUM with two replication and the predicted values and average values of confirmatory experimental results (for *MR* and *R*_*a*_) are also tabulated in Table 9. The confirmatory results for *MR* and *R*_*a*_ have been found to differ from predicted values by 3.42% and 3.14% respectively which are found within the 95% confidence interval (CI).

#### Multi-response optimization using MOPSO

Multi objective evolutionary algorithm produces a Pareto front for the multi-objective minimization problem, which can find out a trade-off solution between conflicting objectives. The Pareto front is defined as the set of non-dominated solutions, where each objective is considered as equally good. A problem can be expressed in terms of a Pareto front multi-objective optimisation problem. From this standpoint, given two solutions s and s′, s′ dominates s if and only if relevance (s′) > relevance(s) and |s′| <|s|. However, if relevance (s′) > relevance(s) but |s′| >|s|, neither solution can dominate the other. The collection of all non- dominating solutions constitutes a surface called Pareto front. The Pareto front consists of those solution for which there exists no better solution in both criteria . Using Pareto front optimisation for a selection problem, there is no need for any a priori assumptions about the importance of objectives^33^ .

**Figure 18:**
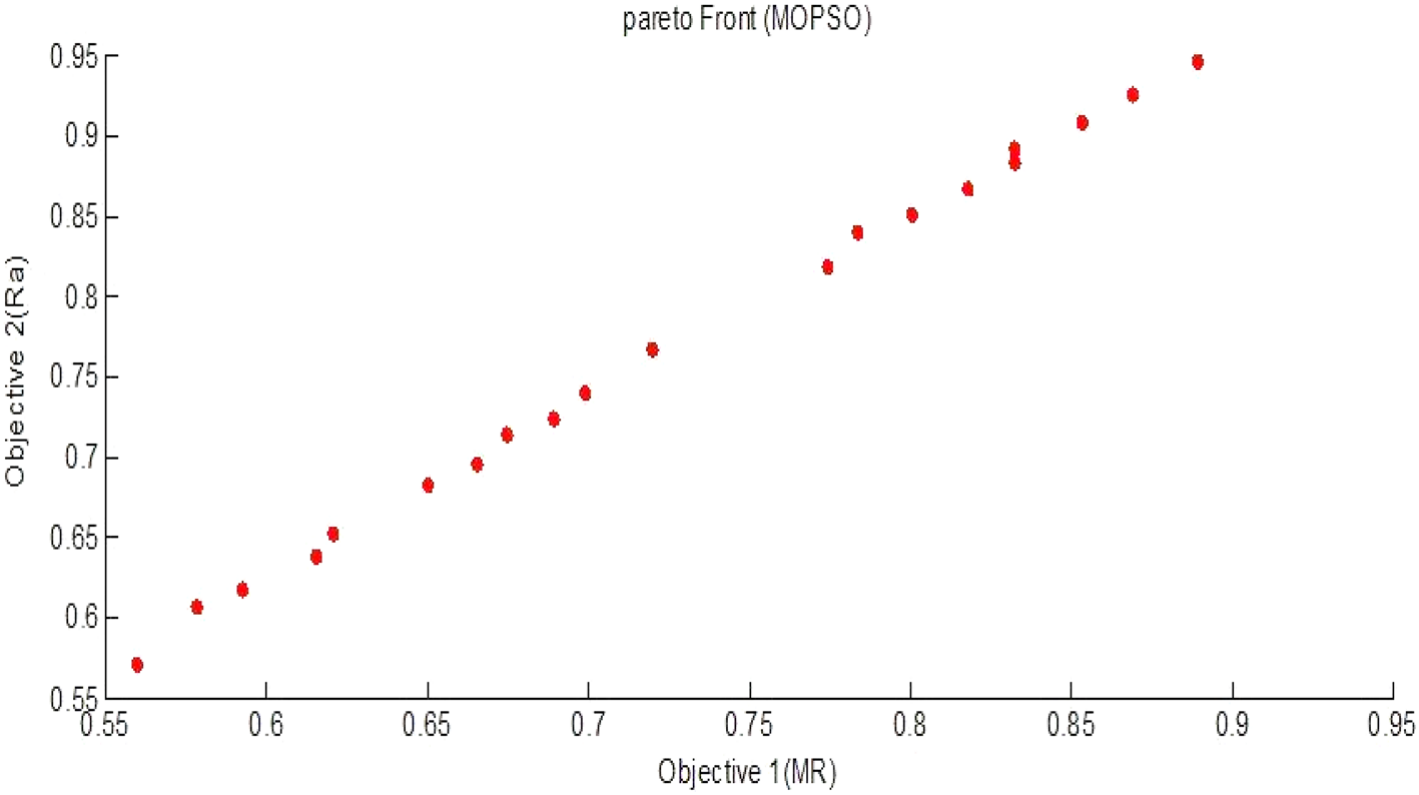
Pareto front for objective functions *MR* and *R*_*a*_*.*

The crowding distance based MOPSO algorithm (In "Optimization through particle swarm optimization" section) is also employed for obtaining the optimized values of process parameters for MR and Ra. The empirical models based on Eq. (2) and (3) both are used for getting the optimized values of process parameters using MOPSO. The pareto front for objective functions MR and R_a_ is shown in Fig. 18. The Pareto front is the set of all Pareto efficient solutions. In muti objective optimization, a large number of solutions are generated as tabulated in Table 10. The solutions are used for getting the best values of process parameters for obtaining maximum values of MR and minimum values of *R*_*a*_ at optimized process parameters. For the confirmation of the results obtained by MOPSO and finding the effectiveness of the optimization technique (MOPSO) some confirmation tests (Sr no. 1 and 2) are carried out on the workpiece and the Table 11. shows that the confirmatory results for *MR* and *R*_*a*_ have been found to differ from predicted values by 3.46% and 4.5% respectively which are found within the 95% confidence interval (CI).

**Table 10:** Optimum values of RUM conditions for *MR* and* R*_*a*_*.*

Sr. No.	Tool rotation	Feed rate	Ultrasonic power	Diamond abrasive grit size	*MR*	*R*_*a*_
1	5366	0.0126	60.06	140	0.5602	0.571
2	5369	0.0167	61.038	140	0.8324	0.883
3	5368	0.0147	61.47	140	0.6988	0.740
4	5373	0.0135	61.49	140	0.6207	0.652
5	5372	0.0163	61.80	140	0.8007	0.850
6	5369	0.0144	61.59	140	0.6749	0.714
7	5368	0.0129	61.60	140	0.5786	0.606
8	5374	0.0159	60.39	140	0.7742	0.818
9	5368	0.0170	63.37	140	0.8533	0.907
10	5375	0.0174	69.42	140	0.889	0.946
11	5372	0.0172	66.86	140	0.8693	0.925
12	5374	0.0160	64.43	140	0.7838	0.839
13	5369	0.0140	60.91	140	0.6500	0.682
14	5367	0.0146	60.37	140	0.6892	0.723
15	5367	0.0151	62.32	140	0.7199	0.767
16	5369	0.0167	66.31	140	0.8326	0.892
17	5367	0.0131	60.99	140	0.5925	0.617
18	5367	0.0142	60.15	140	0.6655	0.695
19	5374	0.0165	60.68	140	0.8180	0.866
20	5367	0.0135	60.19	140	0.6156	0.637
21	5366	0.0126	60.06	140	0.5600	0.571

**Table 11:** Confirmation test for MOPSO.

RUM parameters				Predicted value from PSO	Confirmation through experiments	Predicted value from PSO	Confirmation through experiments
Tool rotation (rpm)	Feed rate (mm/sec)	Ultrasonic power (%)	Diamond abrasive grit size (mesh)	*MR*	MR (mm^3^/sec)	*R*_*a*_ (µm)	*R*_*a*_ (µm)
5366	0.0126	60.06	140	0.5602	0.5803	0.571	0.596
5375	0.0174	69.42	140	0.889	0.8701	0.946	0..998

## Conclusion

In the present study, RUM is employed for machining (drilling) of super alloys (Inconel 718) at different process parameters in order to obtain the optimized process parameters using PSO and MOPSO. The following conclusion are drawn from the present study:

- It is observed that empirical models are quadratic in nature for both *MR* and *R*_*a*_. In addition, two interactions are found significant for *MR* and three interactions are found significant for *R*_*a*_.
- The values of *MR* are increased with increase in the feed rate whereas surface roughness is decreased with increase in feed rate. It is owing to enhancement of tool indentation rate. Conversely, the *MR* is decreased with decrease in mesh size whereas the surface finishing is increased with decrease in mesh size.
- It is concluded that tool rotational speed and ultrasonic power do not significantly affect the *MR* as compared to *R*_*a*_.
- It is witnessed by SEM analysis that the material is withdrawn from the workpiece in the form of big chunks and intercrystallite cracks.
- The maximum value of *MR* of 0.8625 mm^3^/sec is obtained for a tool speed of 5400 rpm, a feed rate of 0.0175 mm/s, an ultrasonic power of 70%, and a diamond abrasive grit size of 140 mesh. The minimum *R*_*a*_ of 0.572 µm is observed for a tool speed of 5400 rpm, a feed rate of 0.0125 mm/s, an ultrasonic power of 60%, and a diamond abrasive grit size of 140 mesh.
- In the case of MOPSO, numbers of solutions are generated at the optimal setting of process parameters in order to get the maximum value of *MR* and minimum values of *R*_*a*_.

## Data Availability

The authors confirm that the data supporting the findings of this study are available within the article [and/or] its supplementary materials.
